# MR Vascular Fingerprinting in Stroke and Brain Tumors Models

**DOI:** 10.1038/srep37071

**Published:** 2016-11-24

**Authors:** B. Lemasson, N. Pannetier, N. Coquery, Ligia S. B. Boisserand, Nora Collomb, N. Schuff, M. Moseley, G. Zaharchuk, E. L. Barbier, T. Christen

**Affiliations:** 1 Univ. Grenoble Alpes, Grenoble Institut des Neurosciences, GIN, F-38000 Grenoble, France; 2 Inserm, U1216, F-38000 Grenoble, France; 3 Center for Imaging of Neurodegenerative diseases, Veterans Affairs Medical Centrer, San Francisco, USA; 4 Department of Radiology, University of California San Francisco, San Francisco, CA, USA; 5 Department of Radiology, Stanford University, Stanford, California, USA

## Abstract

In this study, we evaluated an MRI fingerprinting approach (MRvF) designed to provide high-resolution parametric maps of the microvascular architecture (i.e., blood volume fraction, vessel diameter) and function (blood oxygenation) simultaneously. The method was tested in rats (n = 115), divided in 3 models: brain tumors (9 L, C6, F98), permanent stroke, and a control group of healthy animals. We showed that fingerprinting can robustly distinguish between healthy and pathological brain tissues with different behaviors in tumor and stroke models. In particular, fingerprinting revealed that C6 and F98 glioma models have similar signatures while 9 L present a distinct evolution. We also showed that it is possible to improve the results of MRvF and obtain supplemental information by changing the numerical representation of the vascular network. Finally, good agreement was found between MRvF and conventional MR approaches in healthy tissues and in the C6, F98, and permanent stroke models. For the 9 L glioma model, fingerprinting showed blood oxygenation measurements that contradict results obtained with a quantitative BOLD approach. In conclusion, MR vascular fingerprinting seems to be an efficient technique to study microvascular properties *in vivo*. Multiple technical improvements are feasible and might improve diagnosis and management of brain diseases.

While the spatial resolution of MRI does not allow for direct depiction of small cerebrovascular structures (<30 microns), many methods using endogenous or exogenous tracers have been proposed to infer parameters related to blood architecture and function. This includes steady-state or dynamic susceptibility contrast enhanced^1^, dynamic contrast enhanced^2^, arterial spin labeling^3^, and quantitative BOLD imaging^4^,^5^ that provide measurements of microvessel blood volume fraction, average vessel diameter and density, permeability, blood flow, or blood oxygenation. Clinical studies have benefited from these techniques with special attention paid to diagnosis, prediction of disease progression, and assessment of treatment interventions in stroke^6^ and cancer imaging^7^,^8^. Yet, a number of technical issues based on these approaches are still under investigation after almost 20 years of development, such as: (1) quantification remains a challenge; (2) results obtained in normal tissues are difficult to reproduce in pathological environments; and (3) measurements tend to become less consistent as spatial resolution increases. One common complication in all these approaches is the need for analytical models that describe the subvoxel architecture. Highly restrictive assumptions are made on vessel geometry and/or physiological processes in order to derive closed-form solutions. Thus, the validity of such measurements may be limited to a narrow range of the vascular characteristics. The mean vascular geometry used yields and analytical solution valid for an average voxel, often neglecting that pathologies impact considerably the geometrical characteristics of the vascular network.

A new concept called MR fingerprinting has been recently proposed to estimate MR relaxation times^9^ and could overcome the limitations of analytical methods to measure microvascular properties. In the first study proposed by Ma *et al.*, pseudorandom MR acquisitions that lead to complicated signal evolutions or ‘fingerprints’ in every voxel are compared to a database (or ‘dictionary’) obtained from numerical simulations of the same experiment. Finding the closest match to the fingerprint/dictionary in terms of Euclidian distance allows a direct link between parameters entries of the simulations (in case of Ma *et al.*^9^, T_1_, T_2_, proton density, frequency shift) and the imaged voxel itself. We hypothesized in a recent study^10^ that similar tools could be used to analyze variations in the natural temporal evolution of the MR signal in tissue, resulting in quantitative information about the microvascular network at a sub-voxel imaging scale. In our first implementation, we proposed to use the MR signal decay in a spin echo experiment acquired before and after injection of contrast agent as a ‘vascular fingerprint’. We used a numerical tool that simulates the MR signal from a virtual voxel containing blood vessels as input, computes microscopic magnetic fields and water diffusion effects, and eventually derives the expected MR signal evolution. The parameter inputs of the simulations (blood volume fraction [BVf], mean vessel radius [R], and blood oxygen saturation [StO_2_]) were varied to obtain a dictionary of possible signal evolutions. After the pattern matching procedure, we found that the method enabled the creation of high-resolution parametric maps in the human brain showing expected contrast, fine details, and numerical values in gray matter that were consistent with literature reports.

In theory, this approach has three major advantages over analytical models. First, the numerical simulations incorporate multiple complex interactions between the physiological parameters. As such, there is no need to weigh the influence of “nuisance” parameters (e.g., water diffusion, B1 inhomogeneities) in order to improve the estimation of others (e.g., blood volume, vessel diameter). Nuisance parameters can either be measured and incorporated in the numerical model or directly estimated using the fingerprinting approach. Second, the numerical simulations provide a reasonable description of the MR signal modulations over a large range of physiologically relevant input values. In many analytical approaches, experimental conditions have to be subdivided into separate regimes (static dephasing, diffusion narrowing, etc.) to ensure accurate estimates. These regimes are however not fulfilled under several experimental conditions (e.g., during the passage of a bolus^11^). Third, vascular fingerprinting provides a real advantage in tissues with irregular vascular networks and pathophysiological abnormalities. Numerical models can be adapted to these irregular vascular networks and accurate parameter estimates will be obtained as long as the corresponding virtual voxels are incorporated in the database.

In the present study, we tested the advantage of vascular fingerprinting for microvascular quantification under both normal and pathological conditions. Three different rat models of brain tumors (9 L, C6, F98) known to contain irregular microvascular architectures^12^,^13^,^14^ were analyzed. We also studied a stroke model in which the geometry of blood vessels is less affected but blood flow and blood oxygenation have large variations^4^. We studied the effects of increasing the complexity of the numerical models by adding a new dimension to the dictionary (water diffusion), increasing the size of the dictionary by extending the range of parameters accessible, and finally designing special cases where the voxels contain abnormally large blood vessels with preferential orientations. The results were compared to those obtained from conventional analytical MR methods: steady-state susceptibility contrast imaging for BVf and vessel size imaging (VSI)^15^; and multiparametric quantitative BOLD (mqBOLD) imaging for StO_2_ measurements^4^.

## Materials and Methods

The study design was approved by the local institutional animal care and use committee (COMETHS). All animal procedures conformed to French government guidelines and were performed under permit 380820 and A3851610008 (for experimental and animal care facilities) from the French Ministry of Agriculture (Articles R214–117 to R214–127 published on 7 February 2013). This study is in compliance with the ARRIVE guidelines (Animal Research: Reporting *in Vivo* Experiments)^16^. Animals aged 7 weeks at the start of the experiments were obtained from Charles River, France and housed in groups of 3–4 in Plexiglas cages under standard laboratory condition (12 h light/dark cycle with lights off at 7:00 p.m. and controlled temperature in 22 ± 2 °C). Water and standard laboratory chow were provided ad libitum. After the MRI experiment, animals were euthanized by intra-cardiac injection of Pentobarbital 200 mg/kg (Dolhethal, Vétoquinol Inc, France) under anesthesia by isoflurane (IsoFlo, Abbot Laboratories Ltd, Berkshire, UK).

### Animal preparation

For all experiments, rectal temperature was monitored and rats were maintained at 37.0 ± 0.5 °C. Anesthesia was induced by the inhalation of 5% isoflurane (Abbott Scandinavia AB, Solna, Sweden), and maintained throughout all surgical and imaging procedures with 2–2.5% isoflurane through a facial mask in 80% air-20% oxygen. After anesthesia, the tail vein was equipped with a catheter to deliver contrast agents.

#### Tumor models

Bupivacaine (8 mg/kg; Centravet, France) was subcutaneously injected before incision to prevent postoperative pain. Tumor cell inoculation was performed into the right caudate nucleus (coordinates from bregma: AP = 0, ML = 3.5, DV = 5.5 mm). After injection, the burr hole was filled, the skin incision sewed and rats revived in an incubator before returning to the animal facility. The three tumor model preparations were performed as described below. F98 cells were implanted in the brain of male Fisher 344 rats (244 ± 12 g, Charles River, France, n = 25 analyzed). Five μl of cell suspension in serum-free RPMI1640 (Invitrogen, Pontoise, France) medium containing 5 × 10^3^ cells were inoculated. MRI was performed 20 days after tumor implantation. 9LGS cells were implanted in the brain of male Fisher 344 rats (200–250 g, Charles River, France, n = 23 analyzed). One μl of cell suspension in serum-free RPMI1640 medium containing 1 × 10^4^ cells were inoculated. MRI was performed 10 days after tumor implantation. C6 cells were implanted in the brain of male Wistar (200–250 g, n = 26 analyzed). Five μl of cell suspension in serum-free RPMI1640 medium containing 1 × 10^5^ cells were inoculated. MRI was performed 20 days after tumor implantation.

#### Middle cerebral artery occlusion (MCAO) model

Male Sprague Dawley rats (n = 20 analyzed, 396 ± 9 g; Charles River, France) underwent a permanent focal brain ischemia induced by intraluminal occlusion of the right middle cerebral artery (MCA)^17^. Briefly, the right carotid arterial tree was isolated. A cylinder of melted adhesive (length 2 mm; diameter 0.38 mm) attached to a nylon thread (diameter 0.22 mm) was advanced from the lumen of the external carotid artery into the internal carotid artery up to 5 mm after the external skull base. Rats were imaged 60 min after the occlusion.

#### Healthy animals

Male Fisher 344 rats (250–300 g, n = 23 analyzed) were used as controls.

### *Ex vivo* data

Vessel morphology was analyzed on 4 consecutives 20 μm thick frozen brain sections sampled on 8 animals (four 9 L gliomas and four C6 gliomas). Briefly, after fixation and saturation, brain sections were incubated overnight at 4 °C with primary antibodies to anti-type-IV collagen (Southern Biotech, Clinisciences, Montrouge, France, 1/1000) then with horseradish peroxidase labeled extravidin. Colour was developed with 0.5% (w/v) 3,3-diaminobenzidine (DAB), 0.03% (v/v) hydrogen peroxide followed by counterstaining with hematoxylin. The sections were scanned using a slice scanner at 10X using a Zeiss axio scan system. Quantitative analysis was performed using an automatic segmentation with 7 processing steps (subtract background, colour deconvolution, make binary, remove outliers, despeckle binary, close binary, and fill holes binary) using the freely available image-processing program FIJI (an ImageJ distribution; https://fiji.sc/)^18^. Vessel characterization within 2 regions of interests (ROI; tumor and healthy striatum) was performed using Matlab (extraction of minor and major vessels length and vessels area; The MathWorks Inc., Natick, MA, USA). Then metrics from each of the 4 slides per animal were pooled. Finally we computed the BV_histo_ and the VSI_histo_ for both ROIs using formulae previously published^14^.

### Data acquisition

MRI was conducted with a horizontal bore 4.7 T Biospec animal imager (Bruker Biospin, Ettlingen, Germany) with an actively decoupled cross-coil setup (body coil for radiofrequency transmission and quadrature surface coil for signal reception) and Paravision 5.0.1. After second-order shimming, the following MRI protocol was performed:

1. Anatomical T_2_-weighted (T_2_w) images were acquired using a turbo spin-echo MRI sequence (repetition time (TR)/echo-time (TE) = 4000/33 ms, NEX = 2, 31 slices with a field of view (FOV) = 30 × 30 mm^2^, matrix = 128 × 128 and voxel size = 234 × 234 × 800 µm^3^, acquisition duration 4min 17sec.
2. Apparent Diffusion Coefficient (ADC) was mapped using a diffusion-weighted, spin-echo, single-shot echo-planar imaging (EPI) (TR/TE = 2200/33 ms, 8 averages, 5 slices with FOV = 30 × 30 mm^2^, acquisition matrix = 128 × 96, zero-filled to 128 × 128 and reconstructed voxel size = 234 × 234 × 800 μm^3^). This sequence was applied 6 times; three without diffusion weighting and three times with diffusion weighting (b = 800 s/mm^2^) in three orthogonal directions. Acquisition duration was 3 min 31 sec.
3. Relaxometric maps (T_2_ and T_2_*) were acquired using multiple spin/gradient echo sequences for blood oxygenation mapping, as follows: multiple spin-echo 2D (MSME) (TR = 2000 ms, 26 spin-echoes, ΔTE = 12 ms, 5 slices with FOV = 30 × 30 mm^2^, acquisition matrix = 128 × 96, zero-filled to 128 × 128 and reconstructed voxel size = 234 × 234 × 800 μm^3^, acquisition duration 3 min 12 sec); multiple gradient echo 3D (MGE3D) (TR = 100 ms, 15 gradient-echoes, ΔTE = 4.5 ms, 26 slices with FOV = 30 × 30 mm^2^, matrix = 256 × 256 and voxel size 117 × 117 × 200 μm^3^, acquisition duration 6 min 24 sec).
4. Vascular Fingerprints were acquired using a Gradient Echo Sampling of the Free Induction Decay and Spin Echo (GESFIDE) sequence^19^,^20^ (TR = 4000 ms, 32 echoes, ΔTE = 3.3 ms, SE = 60 ms, 5 slices, NEX 1, acquisition matrix = 128 × 96, zero-filled to 128 × 128 and reconstructed voxel size = 234 × 234 × 800 μm^3^, acquisition duration 6 min 24 sec).
5. Ultrasmall superparamagnetic iron oxide (USPIO) particles were injected via the tail vein over about 20 sec (200 μmol Fe/kg body weight; P904^®^, Guerbet, Roissy, France). 3 min after the injection of USPIOs, the post-contrast GESFIDE sequence was acquired.

### Data processing for steady-state microvascular maps

All processing was performed on a voxel-by-voxel basis using custom code developed in the Matlab environment (The MathWorks Inc., Natick, Ma, USA):

*ADC maps* were computed as the mean of the ADCs observed in each of three orthogonal directions.

*BVf and VSI maps* were estimated using the steady-state approach described by Tropres *et al.*^15^. Changes in transverse relaxation rates due to USPIO (∆R_2_* and ∆R_2_) were obtained from gradient-echo and spin-echo signals acquired with the GESFIDE sequence. BVf and VSI were computed using:


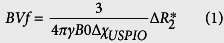



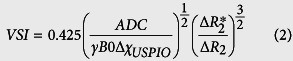


where γ = 2.67502 * 10^8^ rad/s/T, and B0 = 4.7 T. Δχ_USPIO_, the susceptibility difference between blood in the presence and in the absence of USPIO, was set to 3.5ppm (SI units). Note that this value will be different for another type of contrast agent and will change with B0 because the iron oxide particles will be in saturation.

*StO*_*2*_ *maps* were estimated using the quantitative BOLD approach described in refs 4 and 21. Briefly, a T_2_ map was derived from the MSME data using a non-linear fitting algorithm. To correct for the macroscopic B0 inhomogeneities, the MGE3D was spatially averaged as described in refs 4 and 21, leading to an MGE3D dataset with a spatial resolution matching that of the T_2_ and of the BVf maps. The following equation was then fitted voxel-wise to the MR signal decay collected beyond 10 ms echo time of the MGE3D, S(t):


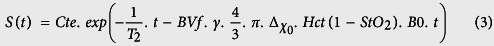


Where ∆χ_0_, the difference between the magnetic susceptibilities of fully oxygenated and fully deoxygenated hemoglobin was set to 3.32 ppm (SI unit) and using T_2_ and BVf from the corresponding maps. Cte is a proportionality constant. Hct, the microvascular hematocrit, was set to 0.42 × 0.85 = 0.357 as described in ref. 21.

### Data processing for MR vascular fingerprinting maps

*Fingerprints* were defined as the signal ratio of the two GESFIDE acquisitions obtained pre- and post-injection of USPIO. Such a fingerprint is sensitive to magnetic inhomogeneity-based dephasing but not to macroscopic B0, B1, and microscopic T_2_ effects^10^. Because of the low signal-to-noise ratio (SNR), the last 8 echoes of the GESFIDE sequences were removed before computing the fingerprints.

#### Numerical simulations

The MR signal was simulated with an iterative numerical approach that models the effects of water protons diffusing in magnetic field gradients produced by the vasculature. We briefly outline here the principal components of the simulation tool, which is described in detail in ref. 22. The voxel contains blood vessels (straight cylinders) that occupy BVf. The lattice inside the simulated voxel is 256 × 256 points, 96 vessels are randomly spread out and the voxel size is adapted to maintain the BVf constraint. The magnetic susceptibility difference, Δχ, between the vessels and the surrounding tissue is given by the StO_2_ according to: Δχ = Δχ_0_.Hct.(1–StO_2_). The magnetic field perturbations inside the voxel are computed using a Fourier-based approach. To speed up the computation, the algorithm is designed in 2D but the magnetic field perturbations are computed in a way that mimics 3D^22^. At each time step δt = 0.5 ms, changes of the magnetization moments are simulated in the lattice using a deterministic iterative approach that models the effects of water diffusion and the rotational effects induced by the magnetic field offset and the RF pulses. The effects of water diffusion are modeled by the convolution of the magnetization lattice with a discrete Gaussian kernel with diffusivity equal to ADC. The MR signal is calculated at each δt by summing the transverse component of the magnetization lattice. Convolutions are performed in the Fourier space to reduce the computational cost. Matlab codes used for the numerical simulations can be found at https://bitbucket.org/NPann/mrvox2d.

#### Dictionaries

Using the numerical tool, we simulated the NMR signal produced by the GESFIDE sequence both with and without contrast agent in the vasculature. The increase in the magnetic susceptibility due to USPIOs (Δχ_USPIO_) was set to 3.5ppm (SI unit) for all voxels and animals. By varying the physiological inputs (oxygenation, water diffusion) and the vascular geometries of the numerical voxels (radius, BVf), we generated 3 dictionaries of simulated vascular MR fingerprints. In Dictionary A: 42 values of BVf were chosen between 0.25 and 25%, 32 values of vessel radius between 0.5 and 100 μm, and 29 values of Δχ between 0 and 1.4 ppm (SI unit, corresponding to StO_2_ = [0–100]%). ADC was kept constant at 800 μm^2^.s^−1^, which corresponds to the average value measured in healthy striatum of all rats (n = 115; 800 ± 85 μm^2^.s^−1^). 77,952 individual signal simulations (38,976 pre and 38,976 post USPIO injection) were required to produce all the numerical fingerprints. The complete list of values is provided in Supplemental Material (Supplementary Tables 1 and 2). In Dictionary B, we extended dictionary A by adding 27 more ADC values between 500 and 1800 μm^2^.s^−1^. This dictionary contains 1,052,352 fingerprints. In Dictionary C, we extended dictionary B by adding voxels that only contained two ‘large blood vessels’ with a specific orientation according to B0. Five values of BVf between 30 and 50%, 8 values of vessel radius between 50 and 1000 μm, and 6 orientations evenly distributed between 0**°** and 90**°** were added. As for dictionary B, 29 values of Δχ and 14 values of ADC were also simulated. 97,440 fingerprints were thus added to Dictionary B. A single signal simulation took about 2.5 s on a desktop computer and the largest dictionary was generated on 30-node cluster in about 24 h.

#### Matching procedure

To increase SNR, a 3 × 3 voxel Gaussian kernel was applied to the *in vivo* data. Fingerprints were calculated voxel-wise and the closest curve in terms of reduced chi-squared was extracted from the dictionaries. The corresponding values of vessel radius, BVf, and StO_2_ were subsequently retrieved to create parametric maps. The goodness of the fit was reported as the coefficient of determination (r^2^). The matching procedure was repeated using the 3 dictionaries independently. For the dictionaries B and C, the ADC values measured from the diffusion-weighted sequence was rounded to the closest simulated ADC value and the corresponding subset of curves was used in the matching procedure.

### Data analysis

#### ROIs

Tumor ROIs were manually delineated on the T_2_w images. For the stroke studies, the ischemic lesion was identified as the hypointense area on ADC maps as proposed previously^23^. The lesion was manually delineated consistently with neuroanatomy (e.g., excluding ventricles and/or small bleeds). Healthy brain was automatically segmented into 3 ROIs: cerebrospinal fluid (CSF), gray matter (GM), and white matter (WM) using a 3D rat brain atlas^24^ and the statistical parametric mapping software (SPM12, Wellcome Department of Cognitive Neurology, London, UK) as described in ref. 24. A striatum ROI was manually delineated in all animals and was used as a contralateral ROI in for lesion animals and as an independent measurement of brain tissue for control animals.

#### Excluded voxels

For the steady-state approach, voxels for which the analysis could not be performed were excluded from the analysis. Exclusion criteria were: voxels for which the fitting did not converge, and voxels with values outside the range of validity of the methods (ADC: [0–3500 μm^2^/s], BVf: [0–17%], VSI: [0–50 μm] and StO_2_: [0–100%]). For the fingerprinting approach, voxels with r^2^ < 0.8 were rejected in the ROI measurement.

#### Statistical analysis

Paired t-tests were used to evaluate for differences between the lesions and contralateral striatum. Repeated measures ANOVA analysis followed by a Bonferroni correction for multiple comparison was used to compare methods (steady-state and fingerprint approach using either dictionary A, B, or C). A p-value less than 0.05 was considered to indicate a significant difference. Statistics were calculated with software (IBM SPSS Statistics v.19, Chicago, IL). Results are presented as the mean ± standard deviation, and are given by group and by ROI.

## Results

Details about the number of animals used, excluded, and analyzed are presented in Supplementary Table 3. n the lesion, 1.2 ± 0.6% of all voxels were excluded from the steady-state BVf/VSI analysis. No voxels were excluded in the healthy striatum. In the steady-state qBOLD approach, 14.6 ± 11.0% voxels were excluded in the lesion and 2.4 ± 1.1% were excluded in the striatum. With the MRvF approach (DictC), 14.2 ± 12.2% of voxels were excluded in the lesion and 2.8 ± 1.5% were excluded in the striatum. The number of excluded voxels was similar between the different dictionaries.

### Study of fingerprints in different tissue types

**Figure 1:**
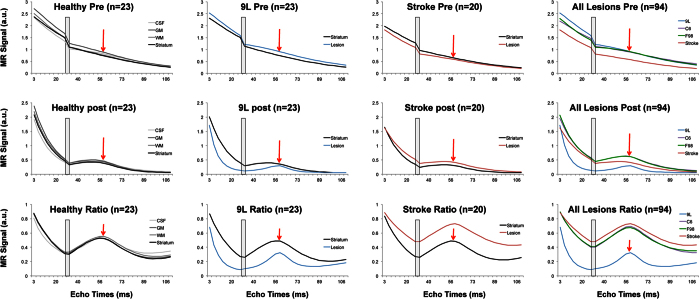
MR signal evolutions obtained using a GESFIDE sequence acquired pre (first row) and post (second row) injection of the contrast agent. The ratio of the signal evolution (pre/post contrast agent injection) is presented in the third row. The signals from 3 groups (Healthy, 9 L, and Stroke for the first, second, and third columns, respectively) are shown in columns. Graphs of the fourth column contain the signal evolution of all lesions. Each curve represents the average of the signal acquired in one group using one ROI. Gray rectangles represent the rapid changes of signals intensities at echo 8 related to the application of the 180 degrees pulse.

MR signal evolutions with echo times, acquired using the GESFIDE sequence, are presented in Fig. 1. One signal corresponds to one ROI and one group averaged over all rats of the corresponding group. Signals pre- and post USPIO (Signal Pre and Signal Post) are shown as well as their ratios, which were used as the actual fingerprints. Rapid changes of signals intensities at echo 8 relate to applications of the 180° pulses and are indicated by gray rectangles. The FID part of the signal measured before contrast injection followed an exponential decay, while the signal after the refocusing pulse does not show a local maximum at the spin echo time (60 ms indicated by a red arrow). This can be explained by the small difference (n.s.) that exists between R2 and R2′ relaxation times in the brain after a careful shimming procedure. Injecting paramagnetic contrast agent increases R2′ which leads to a small signal rebound in Signal Post and a clear spin-echo shape when looking at the ratio of signals. In healthy animals, the signals from all ROIs have similar time evolutions with only small differences (n.s.) observed in the CSF regions. The resemblance between healthy striatum signals in all animals (n = 115; not shown) also suggests reproducibility of the measurements. Indeed, less than 11% of mean value signal variation was observed between all groups and echo times. This is also suggested by observing all the black lines in Fig. 1. Different behaviors can be seen in the lesions ROIs. In the 9 L tumor model, signal dephasing occurs more rapidly than in the corresponding striatum while the opposite is observed in the stroke lesion. The time of the maximum signal rephasing is also different between the lesions and healthy tissues. This can be understood by considering the effects of water diffusion and vessel size on the formation of the spin-echo^25^. The comparison between signal evolution in all lesions suggests that the fingerprints allow distinctions between different pathologies. It is however interesting to note that the signal ratios from C6 and F98 tumors are comparable.

### Match with the dictionaries

**Figure 2:**
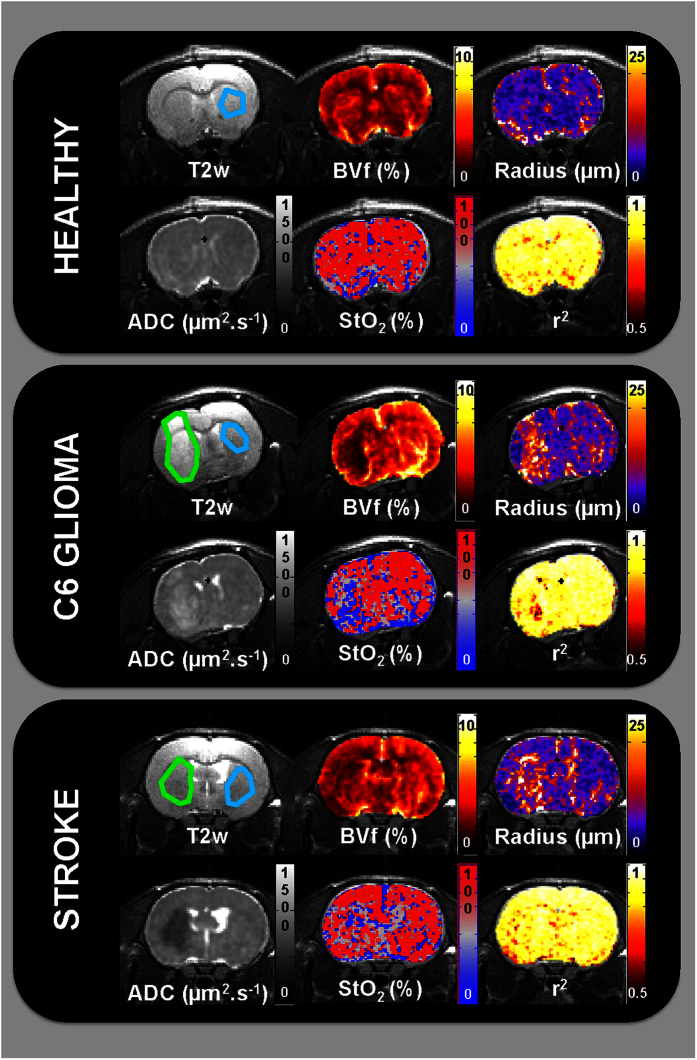
MR images of one representative rat from control, C6, and stroke models. For each animal, T_2_w and ADC images are shown as well as parametric maps obtained with the fingerprinting approach using Dictionary A (fixed ADC value equal to 800 μm^2^.s^−1^). The color-coded parametric maps (BVf, radius, and StO_2_) as well as the map of the coefficient of determination (r^2^) are overlaid on the T_2_w images. Healthy striatum and lesion ROIs are overlaid on the T_2_w images in blue and green, respectively.

Figure 2 shows maps obtained in one representative rat of the following groups: control, C6 glioma, and stroke. In addition to the anatomic T_2_w and diffusion maps, we show the parametric maps of BVf, radius, StO_2_, and r^2^ obtained using the fingerprinting approach and Dictionary A. This includes only one value of ADC equal to 800 μm^2^.s^−1^, which corresponds to the average value measured in healthy striatum of all rats (n = 115; 800 ± 85 μm^2^.s^−1^) and is similar to the dictionary used in the previous human study^10^. While no prior information or initial guess was given during the matching search, we can observe a clear contrast between white and gray matter in the BVf maps of the control animal. Fine details that correspond to larger blood vessels can also be seen. Radius and tissue oxygen saturation maps are more homogeneous. Similar observations were made in the healthy hemispheres of all rats. In the C6 model, the lesion can be observed as a hyperintense signal in the T_2_w and ADC maps. The tumor can also clearly be identified in the parametric maps with lower BVf (3.0 ± 0.7 vs 3.5 ± 0.5%, p < 0.05), higher vessel radius (12.7 ± 3.1 vs 7.3 ± 1.1 μm, p < 0.05), and lower StO_2_ (74.5 ± 4.4 vs 82.5 ± 3.5%, p < 0.05) compared to the healthy striatum. In the permanent stroke model, the lesion cannot be seen in the T_2_w image but shows lower ADC values. In this part of the brain, the BVf and oxygenation are lower than in the healthy hemisphere (BVf: 2.3 ± 0.3 vs. 3.5 ± 0.5%, p < 0.05; StO_2_: 75.7 ± 4.9 vs 82.8 ± 3.7%, p < 0.05, respectively) while blood vessels have larger diameters (15.5 ± 4.2 vs 7.3 ± 1.1 μm, p < 0.05). The r^2^ maps represent the quality of fit between the *in vivo* fingerprint and the corresponding best entry in the dictionary. It can be observed that these values are high (r^2^ > 0.8) for almost all regions in these 3 rats, except a small number of voxels in the C6 tumor. This may correspond to a necrotic area that does not contain any blood vessels or a vessel configuration that has not been simulated in Dictionary A.

**Figure 3:**
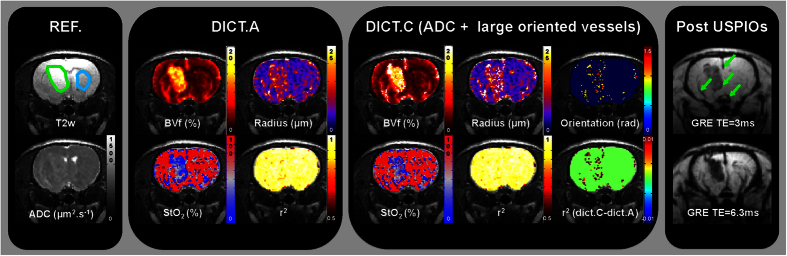
MR images of one representative rat of the 9 L glioma group. The first panel includes T_2_w and ADC images (‘REF’ stands for reference maps). Healthy striatum and lesion ROIs are overlaid on the T_2_w images in blue and green, respectively. The second panel presents the parametric maps obtained with the fingerprinting approach using Dictionary A (ADC value fixed to 800 μm^2^.s^−1^). The color-coded parametric maps (BVf, radius, and StO_2_) as well as the map of the coefficient of determination (r^2^) are overlaid on the T_2_w images. The third panel presents these same parametric maps obtained with the fingerprinting approach using Dictionary C, which includes the ADC map and the simulation of large blood vessels. In addition, a map of the orientation of large blood vessels relative to B0 and a map representing the difference between r^2^ maps obtained using Dictionary C and Dictionary A are shown. The fourth panel shows gradient echo weighted images (GRE) at two different echo times obtained after injection of USPIOs. Green arrows indicate the presence of large blood vessels.

**Figure 4:**
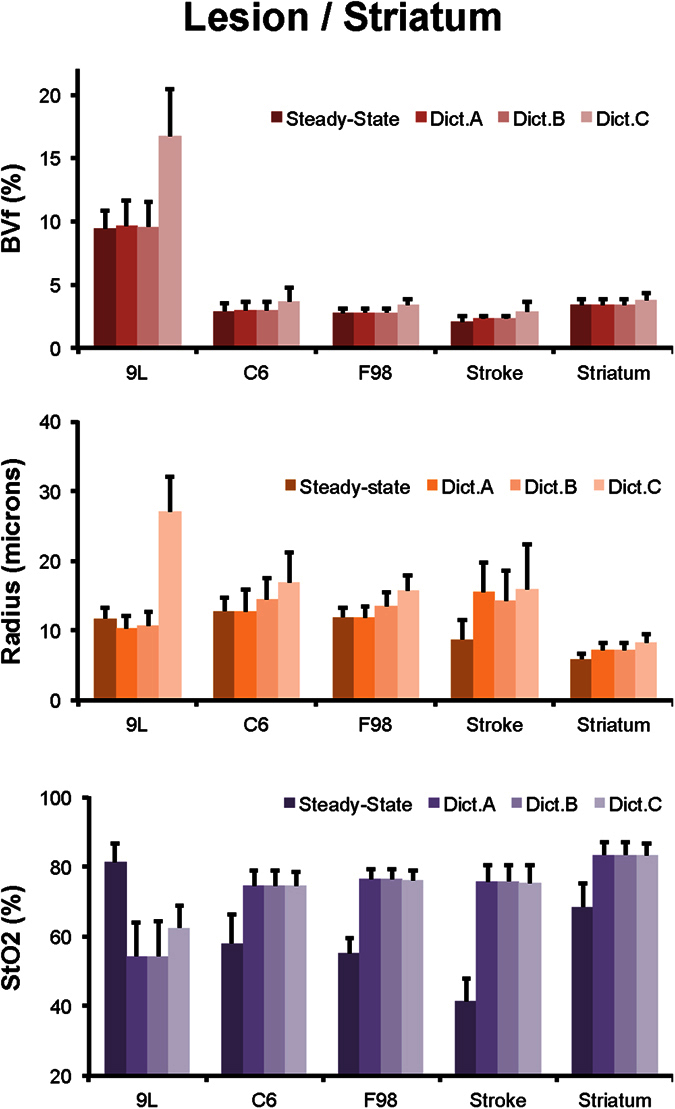
Quantitative estimates of the 3 vascular parameters computed using 4 approaches: the steady-state approach and the fingerprinting approach using the 3 different dictionaries (Dictionaries A, B, and C). For each vascular parameter, bar graphs present the results from the lesion ROIs obtained in the 9 L, C6, F98, and stroke groups as well as the healthy striatum ROI pooled across groups. Data are presented as mean ± s.d.

We compare in Fig. 3 the results obtained with 2 different dictionaries in one rat from the 9 L tumor group. In this glioma model, results from Dictionary A show a clear depiction of the lesion with high blood volume, low tissue oxygen saturation, and a small increase in vessel size. While the match to the fingerprint/dictionary is already high (r^2^ > 0.91), differences can be observed with the results obtained with Dictionary C. These regions mainly involve voxels containing large blood vessels as seen in the post USPIOs images and can be clearly observed in the radius map and in the differential map between r^2^ values. An interesting observation comes from the vessel orientation map that suggests presence of clusters of large blood vessels oriented perpendicular to the main magnetic field in the tumor. Global values for all groups of rats and all dictionaries are summarized in Fig. 4 (graphs for healthy striatum of all rats are provided in Supplementary Figure 1). Only minimal differences were found between the results obtained using Dictionary A and those obtained using Dictionary B (which also includes a search in a hyperplane defined using the measured ADC values). In particular, variations were only observed in the vessel radius measurements in C6, F98, and stroke models. In healthy tissues, changing the dictionary from A to C induces a small but significant increase in blood volume and vessel radius (BVf: 3.4 ± 0.5 vs. 3.7 ± 0.6%; radius: 7.2 ± 1.0 vs. 8.2 ± 1.5 μm for Dict.A vs. Dict.C, respectively; data of each group pooled; p < 0.05). Regardless of the dictionary used, no differences were observed in StO_2_ estimates. It can however be observed that changing the dictionary from A to C has a clear impact on the 9 L model for the 3 vascular parameters (BVf = 9.6 ± 2.0 vs 16.7 ± 3.7%; radius = 10.2 ± 1.9 vs 27.0 ± 5.1 μm and StO_2_ = 54.3 ± 9.7 vs 62.4 ± 6.3%; p < 0.05; Figs 3 and 4). In the 2 other tumor models, changes were observed in the blood volume and vessel size estimates but not on the StO_2_ measurements.

### Comparison with steady-state approaches

**Figure 5:**
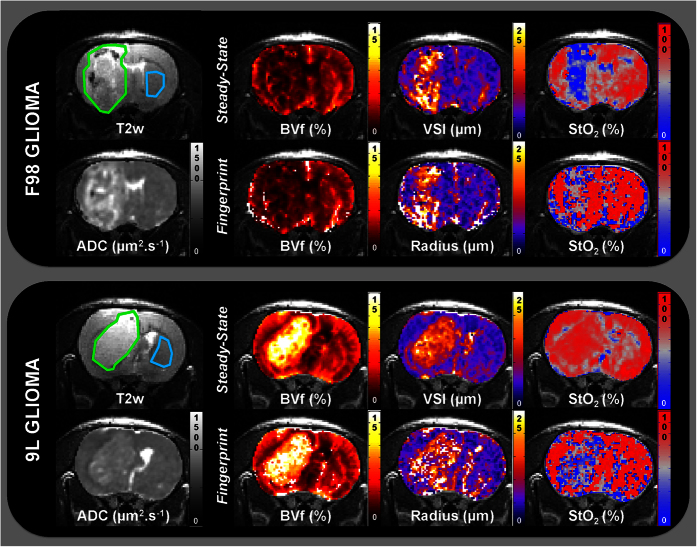
MR images of representative rats from tumor groups F98 (top) and 9 L (bottom). For each animal T_2_w and ADC images acquired are presented as well as parametric maps computed using the steady-state or the fingerprinting (with Dictionary C) approach. Healthy striatum and lesion ROIs are overlaid on the T_2_w images in blue and green, respectively. The color-coded parametric maps (BVf, VSI/radius, and StO_2_) are overlaid on the T_2_w images.

We compared MRvF (with dictionary C) and steady-state approaches in Fig. 5. Visually, the F98 glioma has a higher ADC than in the contralateral striatum. In this tumor model, the steady-state approach shows an increase in VSI and a decrease in BVf and StO_2_ in the lesion compared to healthy tissues (radius = 12.0 ± 1.4 vs. 6.5 ± 0.5 μm; BVf = 2.8 ± 0.4 vs. 3.3 ± 0.4%; and StO_2_ = 54.8 ± 3.5 vs. 70.5 ± 4.5%; p < 0.05; Figs 4 and 5). These trends are also found with the fingerprinting approach except for BVf (no statistical difference was observed between the lesion and the healthy tissue). However, the oxygen values found with MRvF are globally higher than the steady-state estimates. These findings can also be seen in the graphs of Fig. 4 for the entire F98 group and for the C6 and stroke models. In the 9 L animal in Fig. 5, BVf and radius follow the same trend as the steady-state BVf and VSI. However, StO_2_ is higher in the tumor than in the contralateral striatum for the steady-state approach (81.1 ± 5.5 vs. 72.5 ± 6.9%, p < 0.05), while StO_2_ estimates from MRvF are lower in the tumor than in the contralateral striatum (62.4 ± 6.3 vs 83.6 ± 3.4%, p < 0.05). This can also be seen in the graphs of Fig. 4 for the entire 9 L group. A correlation analysis was also performed between MRvF (Dictionary C) and steady-state estimates on an animal level (healthy striatum and lesion ROIs). The results are presented in Supplementary Figure 2. Different colors and symbols are used to represent the different groups of animals. A high linear correlation coefficient (r^2^ = 0.95) was found between the BVf estimates. It has to be noted that the same analysis performed using Dictionary A provided the same correlation coefficient but a trendline with a slope close to one (y = 0.9x+0.2 (Dictionary A) vs y = 0.5x+1.4 (Dictionary C)). This can be understood by the fact that Dictionary C contains medium-to-large blood vessels and larger blood volume fractions, which are not included in the steady-state approach. The results for VSI and vessel radius estimates had lower correlation coefficient (R^2^ = 0.5) and a trendline different from unity. A poor correlation was found between the StO_2_ estimates (R^2^ = 0). However, one can clearly see that the results are heavily influenced by the estimates in the 9 L group (short red bars). When removing the data from the 9 L animals, a larger coefficient (R^2^ = 0.3) was found. In this case, the intercept in the trendline equation (y = 1.1x-27) suggests a bias in the estimates.

### Comparison with *ex vivo* data in two models of tumors

The results of the quantitative *ex vivo* analysis are provided in Supplementary Figure 3. We observed that the mean vessel size estimate (VSI_histo_) and blood volume (BV_histo_) were significantly higher in the 9 L gliomas as compared to both the C6 gliomas and healthy striatum (VSI_histo_: 15.3 ± 1.5 vs. 9.8 ± 2.9 and 7.9 ± 0.6 μm, p < 0.05; BV_histo_ 3.8 ± 0.2 vs. 0.5 ± 0.2 and 0.8 ± 0.4%, p < 0.05, respectively; Supplementary Figure 3). Results are in agreement with the radius and BVf obtained *in vivo* using the fingerprinting approach and Dictionary C but not with the steady-state VSI measurements (Fig. 4).

## Discussion

We showed in this study that it is possible to use a fingerprinting approach to characterize microvascular networks in a large cohort of rats under both normal and pathological conditions. Our choice of fingerprint (spin echo formation pre- and post-contrast) is sensitive enough to distinguish healthy and pathological brain tissues with different behaviors in tumor and stroke models. In particular, we revealed that C6 and F98 glioma models have similar signatures while 9 L present a distinct evolution characterized by the presence of large blood vessels. These observations can be linked to previous observations made with histological measurements and other imaging modalities. Doblas *et al.*^26^ showed using MRI, histology, and immunochemistry that C6 and F98 glioma models do not generate new blood vessels for nutrient supply, but rather the existing vessels grow longer and larger. Conversely, they observed both new blood vessels and altered pre- existing vasculature in the 9 L glioma model.

To validate our approach, we compared MRvF with several MR steady-state techniques. All these methodologies use the same raw signals (FID + spin echo) but differ in terms of post-processing analysis. It is thus easier to evaluate the differences between analytical and numerical models. In healthy tissues, we observed a high correlation between the two types of approaches for BVf mapping. We also found good agreement between the BVf methods in all models of pathology except in the 9 L glioma model where the slope of the correlation line differs from one. This could be explained by the low sensitivity of the steady-state approach to large blood volume fractions. We also observed discrepancies between blood vessel radius obtained with MRvF and steady-state VSI (Supplementary Figure 2). This might be due to the fundamental difference that exists between the averaged vessel radius as measured with MRvF and the vessel size index that is defined as a weighted mean. This finding may be linked to previous comparisons between MRI- and histology-derived estimates of VSI and BVf which reported good correlations between the two methods but also showed that MRI-derived estimates were larger than their histological counterparts by a factor of two^14^. The large difference in vessel radius estimates obtained with the two approaches in the 9 L model (Fig. 4) might also come from the presence of vascular networks that are outside of the mathematical model limits of the steady-state approach. It is indeed assumed in steady-state techniques that the voxels contain a large number of small blood vessels with isotropic directions, and the presence of large vessel structures is neglected. This latter assumption is not met, based on our histological analysis, in which we observed the presence of large blood vessels (>100 μm) in the 9 L model. The quantitative analysis showed that BVf was higher in the 9 L tumor than in the C6 tumor or healthy striatum. These findings were also found with the two MRI approaches. However, histology showed that VSI was higher in the 9 L tumor than in the C6 tumor. This was only found with the MRvF approach (Fig. 4). Lastly, we observed that the tissue oxygen saturation values obtained with the fingerprinting approach were systematically higher than the ones found with the steady-state qBOLD approach. We furthermore showed a substantial difference between StO_2_ estimates in the 9 L glioma model where mqBOLD indicates normal or higher StO_2_ in the tumor, while MRvF indicates lower oxygenation. Previous studies using Electron Paramagnetic Resonance (EPR) have reported normal tissue oxygenation in the 9 L model^27^,^28^. However, other studies using oxygen microelectrodes have reported very low pO_2_ measurements (hypoxic) in the 9 L tumors^29^. It thus appears necessary to further validate MRvF estimates with other independent measurements of brain oxygenation. Larger validation studies using immunohistology or high-resolution intravital microscopy should also be performed. To avoid errors due to spatial registration between modalities, one might also take advantage of the presence of the contrast agent in the vasculature at the end of the protocol to acquire ultra-high resolution MR angiographic images^30^. While the spatial resolution of these acquisitions would not be high enough to allow a direct comparison with MRvF, it would still yield information about the presence of medium-to-large blood vessels and higher blood vessel density. To avoid the large blooming effect due to T_2_* acquisition, a T_1_ based approach would be preferred. Further studies should also evaluate whether the injection itself contributes to the tumoral MRvF (e.g., using an injection of plasma), and assess the response of MRvF estimates to different challenges or therapies.

The vascular fingerprinting approach is likely to benefit from a number of technical improvements. First, we used in our study a basic fingerprint that was known to be sensitive to microvascular characteristics with a theoretical time evolution familiar to MR scientists. Work has to be done to optimize the acquisition parameters such as spin echo time, number of echoes, SNR, and contrast agent dosage for this particular implementation. It could also be interesting to evaluate fingerprinting methods that do not require contrast agent injection, as this would facilitate use in a wider range of clinical environments. The MRvF approach does not need to be restricted to the FID and spin echo signal evolution. Indeed, a great advantage of MRvF is the possibility to choose random acquisition patterns that could maximize the distance between all entries of the dictionary and improve the sensitivity of the approach while reducing acquisition time. To our knowledge, an automatic approach that can design optimal fingerprints for a specific application has not been proposed yet.

After acquiring the signals, we showed that it is possible to improve the results of MRvF by changing the numerical representation of the vascular network. Here we added a new dimension in the dictionaries by simulating the effect of water diffusion and included new vascular geometries. Our first results from histology suggest that this process allowed a better matching with the *in vivo* data and provided supplemental information. One great advantage of our numerical tool is that any type of vascular geometry can be used as an input. In particular, we have already shown that it is possible to use data directly taken from *in vivo* microscopy acquisitions^31^. This could be a great asset for testing the approach in animal models where the data can be directly linked to the anatomical ground truth. Yet, it is clear that this process would require a large number of animals in order to collect enough vascular geometries. A translation to human applications is also not guaranteed of success. To overcome these limitations, one might consider the use of computational methods. For example, it has been shown that vascular growth algorithms^32^, which emulate angiogenesis, can create vascular networks that match measured morphometrics of the cortical microvasculature. Given that these networks are physiologically consistent, oxygen exchange and blood flow can also be simulated^32^. Thus, in the context of cancer studies, MRvF could be eventually linked to mathematical models of tumor growth^33^.

Designing more efficient fingerprints and increasing the size of dictionaries will eventually lead to longer matching procedures. Therefore, efforts have to be made toward reducing post-processing time in MRvF. A recent study^34^ has introduced a fast group-based matching algorithm (GRM) that exploits inherent clustering properties of the dictionaries. The search is then performed in the individual principal component analysis (PCA) space of these subgroups. Using this technique, the authors demonstrated that the search speed was one order of magnitude faster than global PCA and nearly two orders of magnitude faster than direct search. Other authors have proposed to compress the dictionary in the time domain using the singular value decomposition^35^. Using this low-rank approximation, they were able to speed up the pattern recognition algorithm on average by a factor of 4. Kd-trees^36^, binary space-partitioning data structures, could also be considered to complement PC analysis and eventually provide an efficient structure to speed-up the matching process in MRvF.

In conclusion, MR vascular fingerprinting is an efficient technique to study microvascular properties of brain diseases. Multiple technical improvements can be foreseen and might improve diagnosis and prognosis. One can also imagine that a true multimodal approach, where the multiple dimensions used to create the fingerprints are not only obtained with MRI, but also with other imaging modalities such as positron emission tomography or near-infrared spectroscopy, could offer a better vision of brain disorders.

## Additional Information

**How to cite this article**: Lemasson, B. *et al.* MR Vascular Fingerprinting in Stroke and Brain Tumors Models. *Sci. Rep.* **6**, 37071; doi: 10.1038/srep37071 (2016).

**Publisher’s note:** Springer Nature remains neutral with regard to jurisdictional claims in published maps and institutional affiliations.

## Supplementary Material

Supplementary Information
