# Supplementary material for: MR Vascular Fingerprinting in Stroke and Brain Tumors Models
Source: Sci Rep. 2016 Nov 24;6:37071. doi: 10.1038/srep37071 (PMC5121626; doi:10.1038/srep37071)

**MR Vascular Fingerprinting in Stroke and Brain Tumors Models**

**B. Lemasson^1,2^, N. Pannetier^3,4^, N. Coquery^1,2^, Ligia S. B. Boisserand^1,2^, Nora Collomb^1,2^, N. Schuff^3,4^, M. Moseley^5^, G. Zaharchuk^5^, E.L. Barbier^1,2,*^, T. Christen^5^**

^1^Univ. Grenoble Alpes, Grenoble Institut des Neurosciences, GIN, F-38000 Grenoble, France

^2^Inserm, U1216, F-38000 Grenoble, France

^3^Center for Imaging of Neurodegenerative diseases, Veterans Affairs Medical Centrer, San Francisco, USA

^4^Department of Radiology, University of California San Francisco, San Francisco, CA, USA

^5^Department of Radiology, Stanford University, Stanford, California, USA.

**Suppl. Fig 1:** Quantitative estimates of the 3 vascular parameters computed using 4 approaches: the steady-state approach and the fingerprinting approach using 3 different dictionaries (Dict.A, Dict.B, or Dict.C). For each vascular parameter, histograms present the results from the striatum ROIs obtained in the 9L, C6, F98, stroke and healthy groups. Data are presented as mean ± s.d.


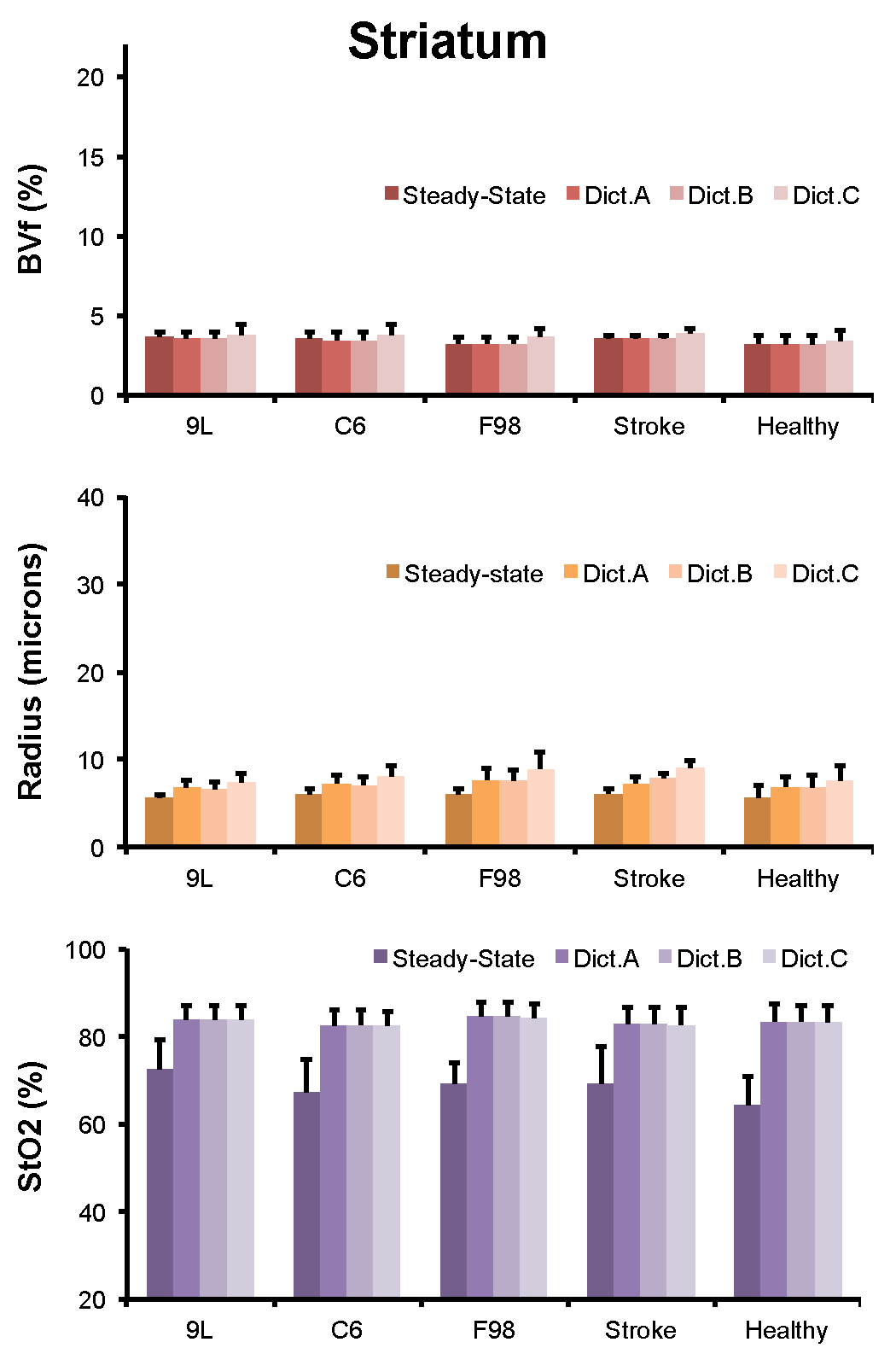


**Suppl. Fig 2:** MR estimates of BVf, VSI and StO_2_ obtained with steady-state approaches as a function of BVf, Vessel radius, and StO_2_ measured with the proposed fingerprinting approach. Data represent the average values obtained in the tumor ROI and contralateral healthy striatum ROI in each rat. Different colors and symbols are associated to the different groups of animals. Linear regression curves as well as corresponding equations and correlation coefficients are given for each scatterplot.


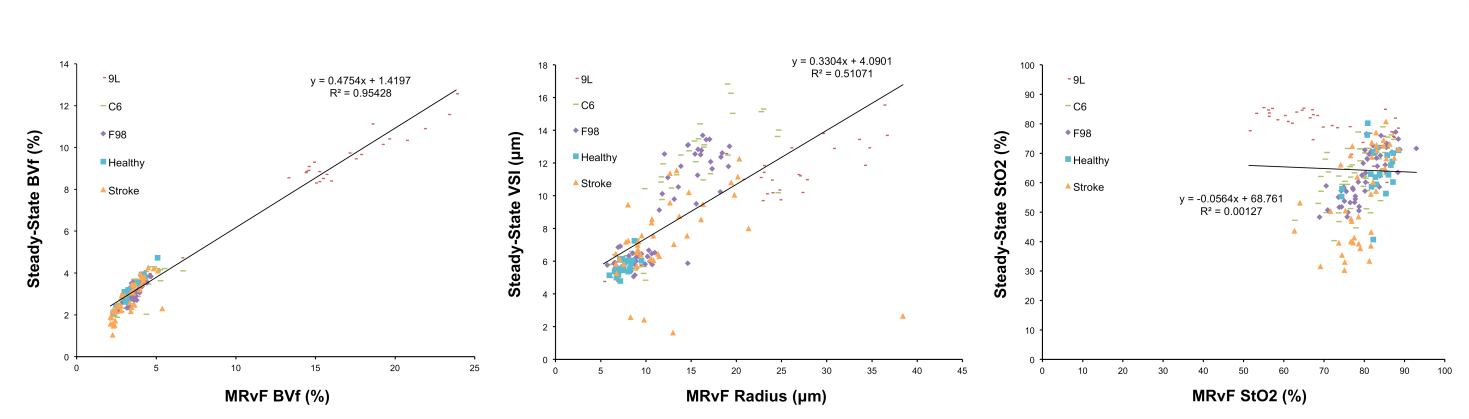


**Suppl. Fig 3:** Quantitative *ex vivo* analysis of the blood volume (BV_histo_) and vessel size (VSI_histo_). Representative images of a 9L glioma (a) and a C6 glioma (b). For each animal, 4 consecutive vessel immunostainings were entirely scanned (at 10x) and vessels were automatically segmented (in yellow). BV_histo_ and the VSI_histo_ were calculated for each animal within the tumor (in red) and the striatum (in green) ROIs across the 4 slices using formulae described by Valable et al.^14^. Mean values of BV_histo_ (c) and of VSI_histo_ (d) were calculated using 4 animals per group and per tumor. For the striatum ROI, data from all animals were pooled (n=8). Data are presented as mean ± SD. * = p<0.05.


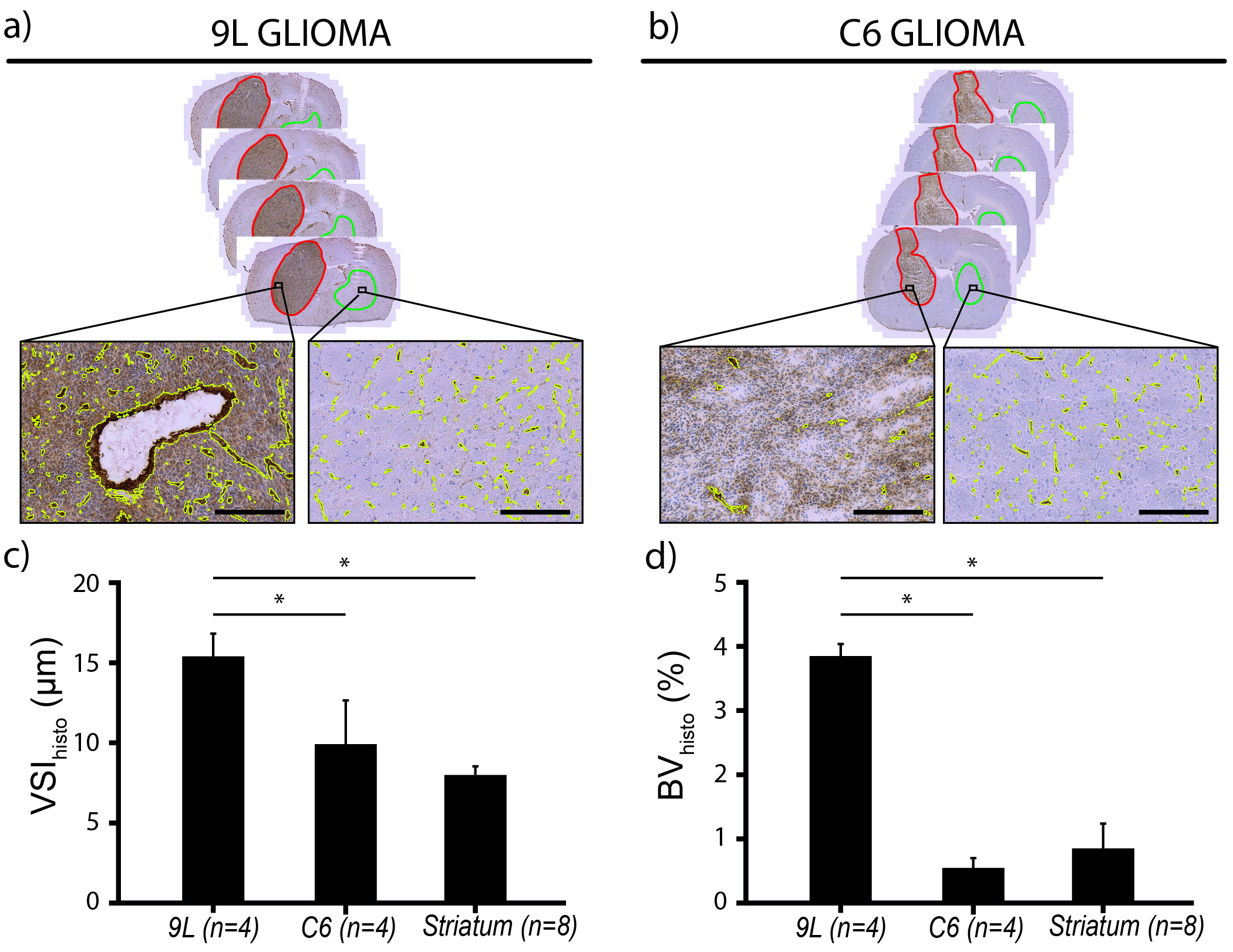


**Suppl. Table 1:** Parameters values for dictionary A and B. Dictionary B contains Dictionary A + a range of diffusion coefficient values (ADC) (shown in bold).

| BVf  (%) | 0.25 | 0.50 | 0.75 | 1.00 | 1.25 | 1.50 | 1.75 | 2.00 | 2.25 | 2.50 | 2.75 | 3.00 | 3.25 | 3.50 | 3.75 |
| --- | --- | --- | --- | --- | --- | --- | --- | --- | --- | --- | --- | --- | --- | --- | --- |
|  | 4.00 | 4.25 | 4.50 | 4.75 | 5.00 | 5.25 | 5.50 | 5.75 | 6.00 | 6.50 | 7.00 | 7.50 | 8.00 | 8.50 | 9.0 |
|  | 9.5 | 10 | 11 | 12 | 13 | 14 | 15 | 17 | 19 | 21 | 23 | 25 |  |  |  |
|  | | | | | | | | | | | | | | | |
| R (μm) | 0.5 | 1.0 | 1.5 | 2.0 | 2.5 | 3.0 | 3.5 | 4.0 | 4.5 | 5.0 | 5.5 | 6.0 | 6.5 | 7.0 | 7.5 |
|  | 8.0 | 8.5 | 9.0 | 9.5 | 10 | 11 | 12 | 13 | 14 | 15 | 17 | 19 | 21 | 23 | 25 |
|  | 50 | 100 |  |  |  |  |  |  |  |  |  |  |  |  |  |
|  | | | | | | | | | | | | | | | |
| Δχ (ppm) | 0 | 0.05 | 0.1 | 0.15 | 0.2 | 0.25 | 0.30 | 0.35 | 0.40 | 0.45 | 0.50 | 0.55 | 0.60 | 0.65 | 0.70 |
|  | 0.75 | 0.80 | 0.85 | 0.90 | 0.95 | 1.00 | 1.05 | 1.10 | 1.15 | 1.20 | 1.25 | 1.30 | 1.35 | 1.40 |  |
|  | | | | | | | | | | | | | | | |
| ADC  μm^2^.s^-1^ | **0.50** | **0.55** | **0.60** | **0.65** | **0.70** | **0.75** | **0.80** | **0.85** | **0.90** | **0.950** | **1.00** | **1.05** | **1.10** | **1.15** | **1.20** |
|  | **1.2** | **1.30** | **1.35** | **1.40** | **1.45** | **1.500** | **1.55** | **1.60** | **1.65** | **1.70** | **1.75** | **1.80** |  |  |  |

**Suppl. Table 2:** Extended parameters values for dictionary C

| BVf  (%) | 0.3 | 0.35 | 0.40 | 0.45 | 0.5 |  |  |  |  | |  |  |  |  |  |  |
| --- | --- | --- | --- | --- | --- | --- | --- | --- | --- | --- | --- | --- | --- | --- | --- | --- |
|  | | | | | | | | | | | | | | | | |
| R (μm) | 50 | 75 | 100 | 150 | 200 | 250 | 500 | 1000 | |  |  |  |  |  |  |  |
|  | | | | | | | | | | | | | | | | |
| Δχ (ppm) | 0 | 0.05 | 0.1 | 0.15 | 0.2 | 0.25 | 0.30 | 0.35 | 0.40 | | 0.45 | 0.50 | 0.55 | 0.60 | 0.65 | 0.70 |
|  | 0.75 | 0.80 | 0.85 | 0.90 | 0.95 | 1.00 | 1.05 | 1.10 | 1.15 | | 1.20 | 1.25 | 1.30 | 1.35 | 1.40 |  |
|  | | | | | | | | | | | | | | | | |
| ADC  μm^2^.s^-1^ | 0.50 | 0.60 | 0.70 | 0.80 | 0.90 | 1.00 | 1.10 | 1.20 | 1.30 | | 1.40 | 1.50 | 1.60 | 1.70 | 1.80 |  |
|  | | | | | | | | | | | | | | | | |
| Dir (°) | 0 | 18 | 36 | 54 | 72 | 90 |  |  |  | |  |  |  |  |  |  |

**Suppl. Table 3:**


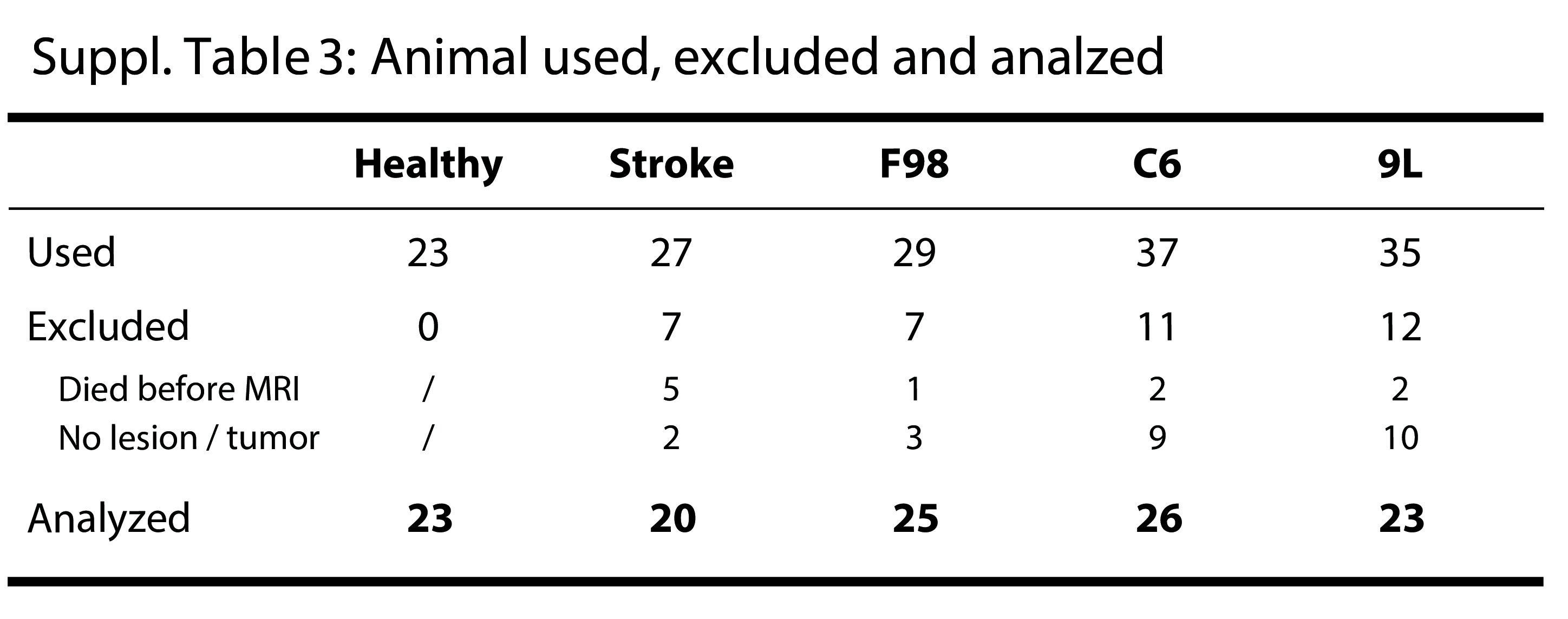

Supplement: Supplementary Information [file srep37071-s1.doc]
